# Hypothalamic S1P/S1PR1 axis controls energy homeostasis in Middle-Aged Rodents: the reversal effects of physical exercise

**DOI:** 10.18632/aging.101138

**Published:** 2016-12-26

**Authors:** Vagner Ramon Rodrigues Silva, Carlos Kiyoshi Katashima, Carla G. Bueno Silva, Luciene Lenhare, Thayana Oliveira Micheletti, Rafael Ludemann Camargo, Ana Carolina Ghezzi, Juliana Alves Camargo, Alexandre Moura Assis, Natalia Tobar, Joseane Morari, Daniela S. Razolli, Leandro Pereira Moura, José Rodrigo Pauli, Dennys Esper Cintra, Lício Augusto Velloso, Mario J.A Saad, Eduardo Rochete Ropelle

**Affiliations:** ^1^ School of Applied Sciences, University of Campinas, Limeira, SP, Brazil; ^2^ Department of Internal Medicine, University of Campinas, Campinas, SP, Brazil; ^3^ CEPECE - Research Center of Sport Sciences, School of Applied Sciences, University of Campinas, Limeira, SP, Brazil; ^4^ Laboratory of Cell Signaling, Obesity and Comorbidities Research Center, University of Campinas, Campinas, 1308-970, Brazil

**Keywords:** aging, hypothalamus, exercise, S1PR1/STAT3

## Abstract

Recently, we demonstrated that the hypothalamic S1PR1/STAT3 axis plays a critical role in the control of food consumption and energy expenditure in rodents. Here, we found that reduction of hypothalamic S1PR1 expression occurs in an age-dependent manner, and was associated with defective thermogenic signaling and weight gain. To address the physiological relevance of these findings, we investigated the effects of chronic and acute exercise on the hypothalamic S1PR1/STAT3 axis. Chronic exercise increased S1PR1 expression and STAT3 phosphorylation in the hypothalamus, restoring the anorexigenic and thermogenic signals in middle-aged mice. Acutely, exercise increased sphingosine-1-phosphate (S1P) levels in the cerebrospinal fluid (CSF) of young rats, whereas the administration of CSF from exercised young rats into the hypothalamus of middle-aged rats at rest was sufficient to reduce the food intake. Finally, the intracerebroventricular (ICV) administration of S1PR1 activators, including the bioactive lipid molecule S1P, and pharmacological S1PR1 activator, SEW2871, induced a potent STAT3 phosphorylation and anorexigenic response in middle-aged rats. Overall, these results suggest that hypothalamic S1PR1 is important for the maintenance of energy balance and provide new insights into the mechanism by which exercise controls the anorexigenic and thermogenic signals in the central nervous system during the aging process.

## INTRODUCTION

The aging process is characterized by the loss of several physiological functions that can lead to diseases [1]. Among these processes, energy balance control stands out, given its role in obesity and metabolic syndromes. It has been demonstrated that aging is related to hyperphagia, a reduction in energy expenditure and increased adiposity in humans and rodents [2-6]. Moreover, brown adipose tissue (BAT) mass appears to decline in human males during aging [7]. BAT is responsible for controlling thermogenesis and energy expenditure through heat production by an uncoupling protein, UCP1 [8]. Defects in UCP1 expression can cause defective thermogenic response, weight gain and metabolic syndrome. In addition, several experimental studies have shown that disruption of hypothalamic function during aging is associated with a reduction in the expression of neuroendocrine hormones, resulting in obesity, responses, an defective thermogenic increased incidence of age-related pathologies, and a reduction in the lifespan of rodents [9-12].

Currently, it has been described that the sphingine-1-phosphate (S1P), a bioactive sphingolipids, is related to cell death as cell migration, proliferation, apoptosis, necrosis and cancer [13-15]. The S1P have five receptors (S1PR1-5) where each receptor has different functions in different tissues [14]. In particular, the S1PR1 receptor, also called EDG1, it has been highlighted that some S1PR1 functions require STAT3 protein. The S1P/S1PR1/STAT3 axis controls the development of several cancer type [16-20]. We described the role of hypothalamic sphingosine-1-phosphate receptor 1 (S1PR1) in the control of energy homeostasis through the Jak/STAT signaling and melanocortin systems in rodents [21]. We reported that neuronal S1P/S1PR1/STAT3 signaling plays an important role in distinct conditions of abnormal feeding behavior, such as obesity and cancer-induced anorexia [21]. The bioactive lipid, sphingosine-1-phosphate (S1P), can activate STAT3 in specific hypothalamic nuclei that control the anorexigenic and thermogenic signals in rodents [21].

Once the physical exercise increases the S1P in plasma levels in humans [22] and also in the muscle [23-25] it seems to be a potent therapeutic target in controlling the maintenance of body weight, food consumption, fat mass and thermogenesis [26-34]. Interestingly, physical exercise also induces upregulation of S1PR1, S1PR2 and S1PR3 in skeletal muscle of rats [25]. Based on these data we hypothesized that defective thermogenic signal and excessive food consumption frequently observed during aging could be, at least in part, associated with the impairment of hypothalamic S1PR1/STAT3 signaling. In addition, we sought to determine the effects of physical exercise on the S1P/S1PR1/STAT3 axis in the hypothalamus of middle-age rodents.

## RESULTS

### Impairment of the hypothalamic S1PR1/STAT3 axis in middle-aged mice

**Figure 1:**
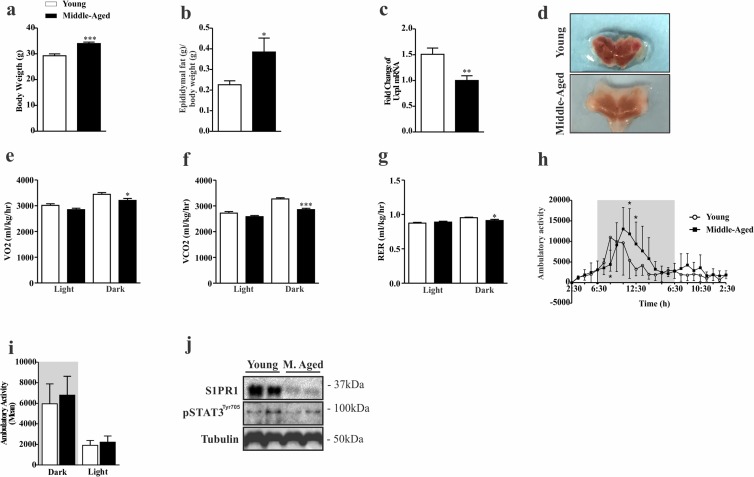
Characterization of phenotypic and evaluation of S1PR1/STAT3 axis in middle-aged mice Body weight **(a)** (n=8 per group) and epididymal fat **(b)** (n=7 per group). UCP1 mRNA levels **(c)** and coloration of BAT **(d)** (n=7–10 per group). The Clams equipment was used to determinate VO_2_ **(e)**, VCO_2_ **(f)**, RER **(g)**, and ambulatory activity point to point during 12 hours in light and dark period **(h)** and mean of cycle light and dark **(i)** (n=8 per group). Western blots showing S1PR1 protein levels and STAT3 tyrosine phosphorylation **(j)** in the hypothalamus of young and middle-aged mice. The mice were fasted prior to hypothalamus extraction for 10 hours (n= 6 per group). The Student's t-test was performed to evaluate data. ± SEM are shown in (a and f) ***p<.0001; (c) **p<0,0021; (b,e and g) *p<0.05. For ambulatory activity point to point, as determined by Student´s t-test where (h) *p<0.05.

First, we sought to determine the pattern of food consumption, body composition, energy expenditure and S1PR1/STAT3 signaling in the hypothalamus of young and middle-aged mice. The results revealed that middle-aged mice have higher body weight and epididymal fat (Fig. 1a, b), decreased UCP1 gene expression (Fig. 1c) and change in the color of BAT (Fig. 1d). In addition, we observed the reduction of consumption VO_2_, production CO_2_ and respiratory exchange ratio (RER) (VCO_2_/VO_2_) (Fig. 1e, f, g) during the dark cycle when compared to younger group. However, ambulatory activity was slightly higher in the middle-aged mice at some points of the dark cycle, when compared to the younger group (Fig. 1h), but no difference was observed when compared to the average of each group (Fig. 1i). Interestingly, these findings were accompanied by a strong reduction of S1PR1 protein levels in the hypothalamus of middle-aged mice when compare to the younger mice. A similar profile was observed in STAT3 phosphorylation (Fig. 1j). These data suggest that hypothalamic S1PR1 signaling could be involved in the control of energy homeostasis during aging.

### Chronic exercise increases S1PR1 in the hypothalamus of middle-aged mice

**Figure 2:**
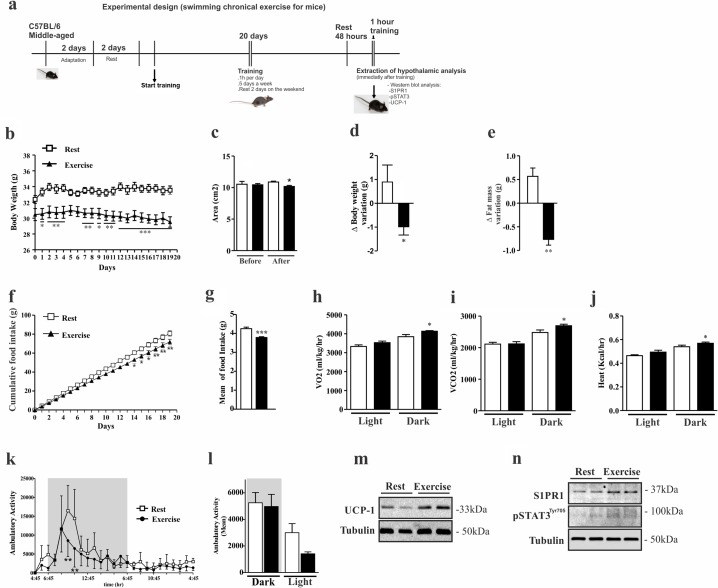
Effects of chronic exercise in the hypothalamus of middle-aged mice Experimental design **(a)**. Body weight curve **(b)**. Dual energy X-ray absorptiometry analyses was performed to evaluate: area **(c)**, body weight variation **(d),** fat mass variation **(e)** (n=3 per group). Cumulative food consumption **(f),** mean of food intake **(g)** (n=11 per group). Clams equipment were used to monitor: O_2_ consumption **(h)**, CO_2_ production **(i)**, heat rate **(j)** and ambulatory activity hours for hours during light and dark periods **(k)** and the mean ambulatory **(l)** in middle-aged exercised and at rest groups (n=3–4 per group). Analyses were made after the last day of training. Western blots of UCP1 protein levels in BAT **(m)**; as well S1PR1 expression and STAT3 phosphorylation in the hypothalamus **(n)** (n=6 per group). The Student's t-test was performed to evaluate data. ± SEM are shown in (e) **p<0.0017; (g) ***p<0.0001; (c,d,h,i and j) *p<0.05. For body weight, cumulative food intake and ambulatory activity point to point, as determined by Student´s t-test where (b) *p<0.05, **p<0.01 and ***p<0.001, (f) *p<0.05, **p<0.01, (k) **p<0.01.

Next, we sought to determine the effects of exercise in the control of S1PR1 signaling in the hypothalamus of middle-aged mice. To address this issue, middle-aged mice were submitted to a chronic exercise (experimental design, Fig. 2a), thereafter we evaluated S1PR1/STAT3 and UCP-1 protein signaling, and monitored food intake, body weight and energy expenditure. First we evaluated the body weight gain between middle-aged mice at rest and exercised animals (Fig. 2b). The Dual energy X-ray absorptiometry (DXA scan) analysis revealed that exercise reduced total body area (Fig. 2c), total body weight (Fig. 2d), and fat mass (Fig. 2e). No difference was found in bone mineral density (suppl. 1a).

Next we analyzed cumulative food consumption and observed a difference between the groups after the second week of training (Fig. 2f); mean daily food intake was lower in the exercised group (Fig. 2g). The chronic exercise increased energy expenditure, through the increase of O_2_ consumption (Fig. 2h), release of CO_2_ (Fig. 2i) and increased heat rate (Fig. 2j). During some points of the dark period, the ambulatory activity was significantly higher in the resting group when compared to exercised group (Fig. 2k). We also observed higher ambulatory activity in the resting group during the light cycle when compared to the exercised group (Fig. 2l).

Next, we evaluated the BAT and hypothalamus from exercised and sedentary middle-aged mice. Western blot analyses showed an increase of UCP1 protein levels in the BAT of exercised mice when compared to rest group (Fig. 2m), confirming that higher energy expenditure occurs in trained mice, as observed. Hypothalamic samples revealed that chronic exercise increased S1PR1 protein levels and STAT3 phosphorylation (Fig. 2n) in the exercised group when compared to resting group.

Since interleukin-6 (IL-6) mediates some anorexigenic and thermogenic responses in the hypothalamus of exercised rodents^20^, we monitored IL-6 serum levels in exercised and sedentary mice. The serum analysis of IL-6 demonstrated high levels of IL-6 in middle-aged mice when compared to the younger group and the physical training appeared to reduce the serum levels of this cytokine (suppl. 1b). Collectively, these data demonstrate that the defective anorexigenic and thermogenic signaling observed during aging could be, at least in part, associated with the down-regulation of hypothalamic S1PR1/STAT3 signaling and the chronical exercise can restore this axis.

### The S1PR1/STAT3 axis is suppressed in the hypothalamus of middle-aged rats

**Figure 3:**
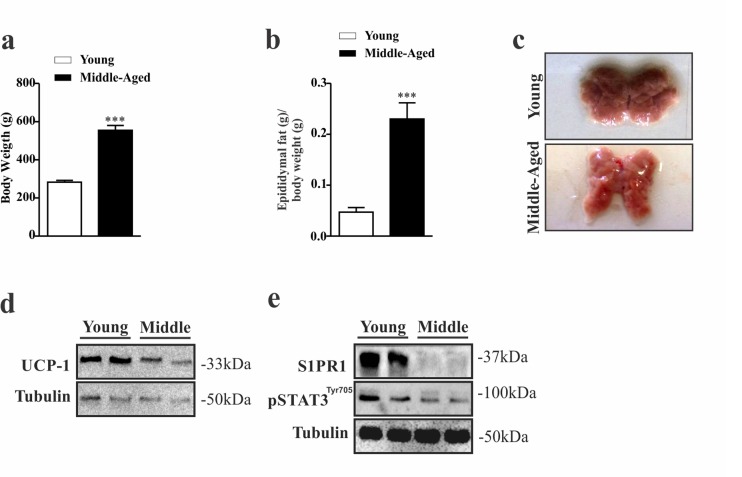
Evaluation of S1PR1/STAT3 axis in the hypothalamus of middle-aged rats Body weight **(a)** (n=6–8 per group) and epididymal fat pad weight **(b)** (n=5 per group). Image of BAT (n=6 per group) **(c)**. Representative Western blots show: UCP1 expression **(d)** S1PR1 protein expression and STAT3 tyrosine phosphorylation in hypothalamus **(e)** in young and middle-aged rats. Rats were fasted for 10 hours prior to extraction of the hypothalamus for Western blots analysis (n=6 per group). The Student's t-test was performed to evaluate data. means ± SEM are shown in (a) ***p<0.0001 to (b) ***p<0.0005.

To confirm our preliminary data, we used another animal model. We evaluated the food intake, body weight, adiposity, BAT and hypothalamus of middle-aged and young Wistar rats. While middle-aged rats displayed higher body weight (Fig. 3a) and epididymal fat (Fig. 3b) compared to younger group, these rats presented alteration in color of the BAT (Fig. 3c) with low protein levels of UCP1 in this tissue (Fig. 3d). Interestingly, these data were accompanied by low levels of S1PR1 expression and STAT3 phosphorylation in the hypothalamus (Fig. 3e). We also compared S1PR1 protein levels and STAT3 phosphorylation in the hypothalamus of middle-aged old rats with those of an advanced age (24 months). We observed a slight reduction of hypothalamic S1PR1 protein in the older rats when compared to middle-aged group, but no difference was observed in the S1PR1/STAT3 phosphorylation between these groups suppl. 2a).

### The hypothalamic S1PR1/STAT3 axis is activated by acute exercise

**Figure 4:**
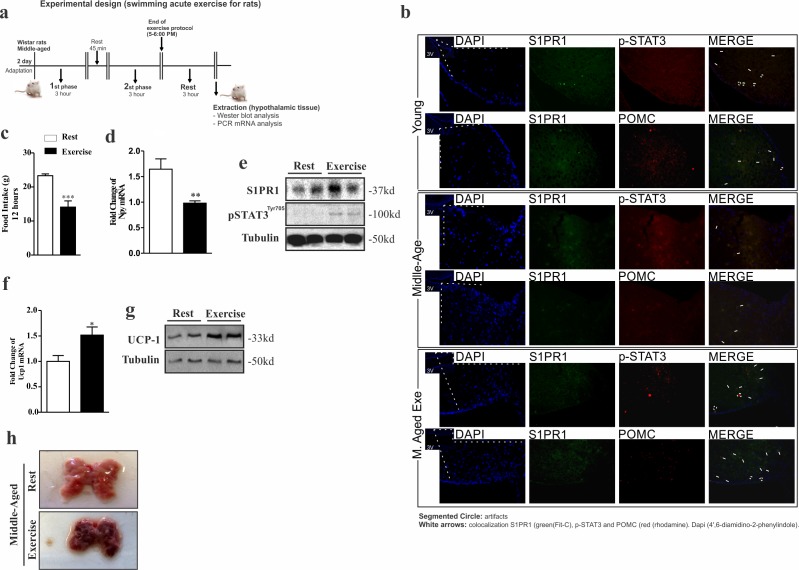
Acute exercise improves S1PR1/STAT3 in the hypothalamus of rats Experimental design **(a)**. Single and double-immunostaining was performed to evaluate the co-localization of S1PR1 (green) with STAT3 phosphorylation and POMC (red) in the third ventricle young, Middle-Aged and Middle-Aged exercised of rats **(b)**. The hypothalamic extraction for analysis was made 3 hours after the acute protocol exercise. Food intake over 12 hours **(c)** (n= 8 per group). Hypothalamic NPY mRNA levels **(d)** (n=6–7 per group). Western blot showing S1PR1 expression and STAT3 phosphorylation **(e)** in the hypothalamus of middle-aged vs. middle-aged exercised (n=6 per group). UCP1 mRNA levels in BAT **(f)** (n=6 per group). Western blot showing UCP1 expression in BAT **(g)** and Image of BAT (n=6 per group) **(h)**. The samples of hypothalamus and BAT were extracted after three hours of exercise. The Student's t-test was performed where (c) ***p<0.0003, (d) **p<0.0026 and (f) *p<0.0123.

Next, we sought to investigate the effect of acute exercise on hypothalamic S1PR1/STAT3/POMC signaling in middle-aged rats (experimental design, Fig. 4a). Initially, we analyzed the relationship hypothalamic neuronal about the S1PR1 receptor between the STAT3 phosphorylation and anorexigenic POMC neuronal in the third ventricle on young, Middle-Aged and Middle-Aged exercised. The results showed interaction between these molecules (Fig. 4b). Next, our results showed that acute exercise reduced food consumption in middle-aged rats (Fig. 4c), which was accompanied by a reduction of neuropeptide Y (NPY) mRNA levels in the hypothalamus (Fig. 4d). Notably, prolonged, acute exercise was able to increase S1PR1 protein levels and STAT3 phosphorylation in the hypothalamus (Fig. 4e) in addition to increasing levels of UCP1 mRNA and its coding protein (Fig. 4f and g). BAT coloration was changed (Fig. 4h). There was no change in total body weight or epididymal fat pad weight between these groups (data not shown).

Importantly, the serum level of IL-6 was higher in middle-aged rats when compared to the young group, but a single session of acute exercise was not enough to increase significantly the serum levels of IL-6 in this experimental group (suppl. 3a).

### Hypothalamic stimulation of S1PR1/STAT3 signaling reduces food intake in middle-aged rats

**Figure 5:**
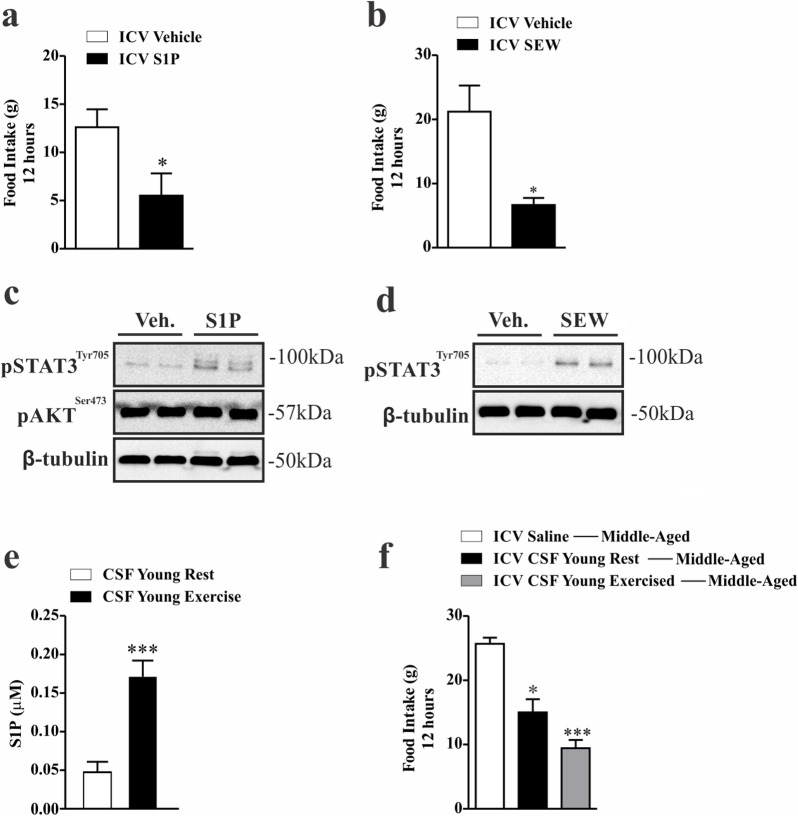
Anorexigenic effects of hypothalamic S1PR1 activation in middle-aged rats 12 hours of food consumption after ICV injection of S1P (50 ng) **(a)** or SEW2871 (50 nM) **(b)** (n=5–6 per group), as analyzed by Student's t-test. Western blot showing, STAT3^tyr705^ and AKT^ser473^ phosphorylation in hypothalamic samples of Wistar rats 30 minutes after S1P injection **(c)** (n=6 per group). Western blot showing STAT3^tyr705^ phosphorylation. The samples were obtained 30 minutes after ICV SEW2871 injection **(d)** (n=6 per group). S1P levels (μM) of CSF in young rats at rest and after acute exercise, as analysed by Student's t-test **(e)** (n=5 per group). Food intake **(f)** for 12 hours in middle-aged rats after ICV injection of CSF (2 μL) from young rats at rest and from young exercised rats in middle-aged rats (n=6–7 per group). The Student's t-test where (a) *p<0.0229, (b) **p<0.0022, (e) ***p<0.0006. One-way ANOVA was used to (f) *** p<0.0001 vs ICV Saline Middle-Aged and ICV CSF Young Rest; *p<0.05 vs ICV CSF Young Exercised ---Middle-Aged.

To determine if specific stimulation of S1PR1 in the hypothalamus modulates energy homeostasis in middle-aged rats, we performed an acute intracerebroventricular (ICV) injection of two different S1PR1 agonists. We observed that both agonists, S1P and SEW2871, promoted a strong reduction in food consumption (Fig. 5a and b) which was accompanied by high levels of STAT3 phosphorylation in the hypothalamus of middle-aged rats (Fig. 5c and d). It was not found difference in body weight (data not shown). No difference was found in the levels of hypothalamic Akt phosphorylation after S1P injection (Fig. 5c), suggesting that S1PR1 induces the anorexigenic signal using leptin but not canonical insulin signaling.

However, one could argue that the anorexigenic effects observed after pharmacological stimulation of hypothalamic S1PR1 could be related to the non-physiological concentration of S1P, and/or by the toxicity mediated by SEW2871 in the central nervous system. To address this issue, we obtained the physiological levels of S1P from the cerebrospinal fluid (CSF) of young rats at rest and immediately after the acute exercise protocol. Surprisingly, we first observed that this acute exercise markedly increased S1P levels in the CSF of young rats (Fig. 5e). When the CSF of young, at rest mice was injected into middle-aged rats, we observed reduced food consumption by about 50% in middle-aged rats. Thereafter, we used the CSF from exercised rats with very high levels of S1P, and we observed that the ICV injection of CSF from exercised animals into middle-aged rats promoted a strong anorexigenic effect (Fig. 5f), suggesting that physiological concentrations of S1P mediates a potent anorexigenic signal.

## DISCUSSION

In the present study, we investigated the role of the S1P/S1PR1 axis in the control of energy homeostasis of middle-aged rodents. Initial findings revealed that middle-aged rats had higher adipose levels compared to younger rats, as well as a strong reduction in energy expenditure, low UCP1 expression and change in the color of BAT. BAT plays a key role in thermogenesis and energy homeostasis in rodents and humans, through UCP1 [2-4]. Several studies have shown that low expression of UCP1 can cause several metabolic diseases such as obesity, type II diabetes and diseases related to age [3, 8]. Furthermore, it was shown that the activity and mass of BAT are higher in younger people or female individuals. Strong evidence reports that the impact of activity, BAT mass and consequently increased adiposity are diminished in the elderly [7]. In addition, while this decrease was found in females with increasing age, the strongest effects were found in older males [7].

A decrease in the consumption of O_2_, release CO_2_, and RER during the dark cycle were also observed in middle-aged rats. A reduction in RER suggests that this group consumed more carbohydrates and fat, which were used as an energy [35]. We also observed a robust reduction in S1PR1 protein levels and STAT3 phosphorylation in the hypothalamus of two different, middle-aged animal models. Taken together, these data suggest that, at least in part, defective anorexigenic and thermogenic signaling in middle-aged rodents could be associated with an impairment in hypothalamic S1PR1/STAT3 signaling.

S1P synthesis occurs by phosphorylation of sphingosine, a reaction which is catalyzed by the sphingosine kinases, SphK1 and SphK2. S1P mediates its biological effects via paracrine or autocrine mechanisms [14]. Under pathological conditions, Liang *et al*. showed that aberrant levels of S1P occurs through up-regulation of SphK1 in cancer cells, which contributes to inflammation and the progression of colon cancer [17]. Moreover, this change in S1P synthesis resulting in high serum S1P levels was observed in obese animal models [36, 37] as well as in obese individuals [38]. Based on these studies, we postulate that exercise could be a strong physiological modulator of S1P/S1PR1/STAT3 signaling, and that modulation of this axis can occur through two different mechanisms. The first is that acute exercise can increase circulating S1P levels, the second is that chronic exercise can restore hypothalamic levels of S1PR1 in middle-aged mice. However, the mechanisms by which exercise modulates S1P levels and hypothalamic S1PR1 expression are unknown and deserves further investigation.

Many aging-related diseases are linked to inflammatory processes [39-42]. Consistent with this notion, IL-6 an inflammatory cytokine has been reported to cause human prostate cancer, and is associated with morbidity and mortality when there are increased levels [42-46]. In the present study, chronic physical exercise was able to reduce circulating levels of IL-6 when compared to the sedentary middle-aged group. Conversely, acute exercise increased the IL-6 levels without statistical significance. These data led us to suspect that other circulating factor(s) are related to the anorexigenic and thermogenic effects observed in exercising middle-aged rodents. It is important to mention that chronic and acute effects of exercise should be analyzed in distinct manners, as chronic exercise promotes several physiological adaptations, including changes in body composition, hormonal profile, anti-inflammatory response, and others [47, 48]. These significant alterations in the metabolism make the interpretation of some results difficult. For this reason, we decide to use an acute exercise protocol. The utilization of two different exercise protocols showed that chronic exercise had a robust effect on S1PR1 protein levels in the hypothalamus, when compared to acute exercise; however, acute exercise was sufficient to stimulate S1P levels.

The effects of exercise on the control of food intake is a complex phenomenon and depends on many circumstances, including intensity, duration, type of exercise and especially the individuals enrolled in the exercise program. Recent studies have shown that exercise suppresses appetite in overweight and obese individuals [49-52], including adolescents and postmenopausal women [53]. However, the mechanism by which exercise modulates energy consumption is not clear. Other studies demonstrated that long term exercise induces the secretion of biomolecules derived from muscle including interleukins [54], apelin [55], acids and proteins rich in cysteine [56], and irisin [32] that promote the beneficial effects of exercise.

Baranowske *et al*. demonstrated that acute exercise increases the level of S1P in plasma and erythrocyte ceramides of untrained subjects [22], while Banitalebi *et al*. showed that resistance training induces an increase in plasma levels of S1P with overexpression of its receptors S1PR1, S1PR2 and S1PR3 in the skeletal muscle of rats [25]. It appears that the plasma concentration of S1P only reaches high levels after performing high-intensity exercise, through increased production of sphingosine by skeletal muscle in humans [24]. There is some evidence supporting a key role of NPY in the control of hunger and energy expenditure during aging [57,58]. Here, we demonstrated that the physical exercise elicited the S1P/S1PR1/STAT3 axis, reduced NPY mRNA levels and that this phenomenon was associated with the energy intake and energy expenditure pattern observed in exercised middle-aged rats. The present study reports high levels of S1P in the CSF of young, exercised rats. When injected with CSF from either young rats at rest, or exercised young rats in middle-aged rats, noted that the anorexigenic effect it was increased, we believe that this are related to the amount of S1P contained in the CSF. However, it is important to note that high levels of S1P were found in hyperphagic mice, *ob/ob* [36]. This paradox can be explained by the strong downregulation of S1PR1 expression observed in the hypothalamus of obese rats when compared to a lean group [21]. Likely, low levels of S1PR1 protein in the hypothalamus of obese animals is associated with deficient STAT3 signaling [59, 60], since STAT3 is a direct transcriptional activator of the promoter *S1pr1* [20]. Interestingly, the expression of STAT3/S1PR1 as the S1P/S1PR1 axis is important for persistent phosphorylation of STAT3 results in a positive feedback circuit [20]. Thus, we believe that this similar mechanism occurs during aging, since the aging process leads to defective hypothalamic STAT3 activity [58].

Upregulation of S1P levels in the CSF of exercised animals appears to be sufficient to normalize S1PR1 protein levels in the hypothalamic tissue of aged rodents. S1PRs are composed of five subunits (S1PR1-5) which activate several distinct signaling pathways in response to S1P [14]. Recently, we reported high levels of S1PR1 in the hypothalamus compared to various peripheral tissues. Importantly, S1PR1 and STAT3 are expressed in the same neuronal cells, confirming that crosstalk between S1P and leptin signaling occurs [21]. This information suggests that S1PR1 induces anorectic effects through STAT3 activity, since STAT3 controls the expression of anorexigenic in hypothalamic neurons [61]. In the present study we demonstrated that acute ICV injection of S1P induced STAT3 phosphorylation in the hypothalamus and reduced food intake in middle-aged rats. Although S1P induced potent STAT3 phosphorylation, S1P is not a specific S1PR1 activator. Thus, we performed an additional set of experiment by using a specific S1PR1 agonist (SEW2871), confirming the role of hypothalamic S1PR1 on STAT3 phosphorylation.

Lastly, our study showed that impairment of the S1PR1/STAT3 axis in the hypothalamus of middle-aged rodents was linked to defective anorexigenic and thermogenic signaling. In addition, we also show that physical exercise can increase S1P levels and restore the S1PR1/STAT3 hypothalamic in middle-aged rodents, increasing energy expenditure and food consumption.

## MATERIALS AND METHODS

### Animals and diet

Wild-type mice (C57BL/6) and Wistar rats male were used. Mice were either young (2 months), or middle-aged (12–15 months), and only old rats (24 months) were used. All animals were obtained from the University of Campinas Breeding Center. The project was approved by the ethics committee of the University of Campinas (number: 3137-1), which follows the international university guidelines for the use of animals in experimental studies and experiments. All animals were maintained in a 12:12 hour light and dark cycle, and housed in cages between 22–24°C. The light cycle started at 6:00 am. The animals were fed with a standard diet (3.948 kcal·kg^−1^) ad libitum.

### Intracerebroventricular Cannulation and Injection (ICV)

Stereotactic surgery in Wistar rats was done as described previously [21]. *S1P and SEW2871 injection*: Rats were fasted for 10 hours prior to injection. For Western blot analysis, ICV of S1P (50 ng) and SEW (50 nM) or respective vehicles were injected into the third ventricle. To evaluate the effects of S1P and SEW on STAT3 phosphorylation signaling, the hypothalamic tissue was removed 30 minutes later. To evaluate the effects of S1P and SEW on food intake and body weight in rats, ICV injections were all performed between 5:00–6:00 p.m. *CSF injection*: CSF was obtained from Wistar rats at rest or immediately after acute exercise through the introduction of a needle into the cisterna magna through the skin and/or dura mater using a stereotactic micromanipulator, as previously described [62] with minor modifications. Immediately after the liquor sampling, 2 μL of CSF or vehicle (saline) was injected into the third ventricle of middle-aged rats to evaluate food intake and body weight during 12 hour period. CSF injections were performed between 5:00–6:00 p.m.

### Chronic exercise protocol (for mice)

First, the mice were acclimated to swimming for two days, at ten minutes per day. Water temperature was maintained at 32°C. The mice swam in groups of four in plastic barrels of 40 cm in diameter that were filled to a depth of 20 cm for one hour, during 5 days per four weeks. Cumulative food consumption and body weight measurements were made every day during the exercise protocol. Extraction of hypothalamic tissue was made after last (20^th^) session of the exercise protocol.

### Acute Exercise protocol (for rats)

The Wistar rats exercised for only one session of acute swimming, as described previously [63]. The acute exercise protocol for rats were finished between 5:00–6:00 p.m, for evaluation of food intake and body weight per 12 hour period and analysis of hypothalamic tissue. Samples of hypothalamic tissue were extracted after three hours of acute exercise.

### Dual energy X-ray absorptiometry (DXA scan)

The mice were anaesthetized. The mensuration of body composition was evaluated in mice before and after chronical exercise protocol by Version 13.3:5 Rat Whole Body. Model: Discovery Wi (S/N 83901) by HOLOGIC Located in the Nuclear Medicine Service in the Hospital das Clínicas - Unicamp / Cidade Universitária Zeferino Vaz. SP-13083-888.

### BAT Imaging

Images were taken immediately after extraction of BAT.

### Oxygen consumption determination

The mice were acclimated for 24 hours to an open circuit calorimeter system, the LE405 Gas Analyzer (Panlab – Harvard Apparatus, Holliston, MA, USA), which was calibrated as recommended by the company and used to measure the rate of O_2_ consumption, CO_2_ production_,_ RER, heat rate and ambulatory activity during the light and dark periods. Data was recorded for 24 hours, and analyses were made between: young vs middle-aged mice, and middle-aged sedentary vs middle-aged exercised mice.

### Determination of S1P and IL6 levels

*For mice*: Blood was collected from the cava vein following the last (20^th^) session of chronic exercise for mice. Plasma was separated by centrifugation (1,100 x g) for 15 minutes at 4°C and stored at −80°C until the assay. IL-6 concentrations were determined using a commercially available ELISA for mice, following the manufacturer's instructions. *For rats*: three hours after acute exercise protocol, blood was collected from the cava vein. Plasma was separated by centrifugation (1,100 x g) for 15 min at 4°C and stored at −80°C until the assay. IL-6 concentrations were determined using a commercially available ELISA for rats, following the manufacturer's instructions. CSF was obtained from young male rats at rest and exercised, and was extracted immediately after acute exercise protocol through the cisterna magna puncture using the stereotaxic apparatus. For serum and CSF S1P determination, we employed a commercially available S1P assay kit (Echelon Biosciences Inc.)

### Western blot analyses

Three hours after acute exercise for rats, the hypothalamus was extracted. The animals were anesthetized and the hypothalamus quickly removed, minced coarsely, and homogenized immediately in a freshly prepared ice-cold buffer (1 % Triton X-100, 100 mmol/L Tris pH 7.4, 100 mmol/L sodium pyrophosphate, 100 mmol/L sodium fluoride, 10 mmol/L EDTA, 10 mmol/L sodium vanadate, 2 mmol/L phenyl methylsulphonyl fluoride, and 0.1 mg aprotinin) suitable for preserving the phosphorylation state of enzymes. Proteins were separated by SDS-PAGE and transferred to nitrocellulose membranes. Specific antibodies (see below) and SuperSignal^TM^ West Pico Chemiluminescent Substrate (Thermo Scientific) were used for protein analyses. The membranes uncut you can see in supplementary 4.

### Immunohistochemistry

Paraformaldehyde-fixed rat hypothalami were sectioned (5 mm). The sections were obtained from the hypothalami of four rats per group in the same localization (antero–posterior = −1.78 from bregma) and were subjected to regular single- or double-immunofluorescence staining using 4′,6-diamidino-2-phenylindole, anti-EDG1(S1PR1), anti-pSTAT3, anti-POMC. Analysis and photo documentation of results were performed using a LSM 510 laser confocal microscope (Zeiss, Jena, Germany). Anatomical correlations were made according to the landmarks given in a stereotaxic atlas.

### Antibodies and chemicals

Anti-EDG-1 (rabbit polyclonal, SC-25489), anti-UCP1 (goat polyclonal, M-17: SC-6529) antibodies were from Santa Cruz Biotechnology, Inc. anti-β Tubulin (rabbit polyclonal, #2146) anti-phospho-Akt (rabbit polyclonal, 9271s) and anti-phospho-STAT3 (rabbit polyclonal, #9131), were from Cell Signaling Technology (Beverly, MA, USA). The antibody solution was 1:1000 for Western blots. Protein A-Sepharose 6 MB and nitrocellulose paper (Hybond ECL, 0.45 mm) were from Amersham Pharmacia Biotech United Kingdom Ltd. (Buckinghamshire, United Kingdom). S1P was from Avanti Polar Lipids Inc. (Alabama, EUA) and SEW2871 was from Cayman Chemical (Michigan, USA).

### mRNA Isolation and Real Time PCR

Total hypothalamic RNA was extracted using Trizol reagent (Life Technologies, Gaithersburg, MD, USA), according to the manufacturer's recommendations. Total RNA was isolated through DNA digestion with RNase-free DNase (RQ1, Promega, Madison, WI, USA). The BAT of mice was extracted after the last (20^th^) session of exercise for real time PCR analysis. Real time PCR and mRNA isolation were performed using commercial kits, following the manufacturer's protocols: UCP1: Mm01244861_m1 and Mouse GAPD (GAPDH) Endogenous Control (Catalog number: 4352339E).

The BAT and hypothalamus of rats were extracted after three hours exercise for real time PCR analyses. Real time PCR and mRNA isolation were performed using commercial kits, as follows: UCP1: - Rn00562126_m1, NPY: Rn00561681_m1 and Rat GAPD (GAPDH) Endogenous Control - Catalog number: 4352338E

### Statistical analysis

All statistics were performed when necessary using the Student's t test, with the Bonferroni posthoc test. Significance was established at the p<0.05 level.

## SUPPLEMENTARY MATERIALS FIGURES



## References

[1] Miller RA (2012). Genes against aging. J Gerontol A Biol Sci Med Sci.

[2] Tchkonia T, Morbeck DE, Von Zglinicki T, Van Deursen J, Lustgarten J, Scrable H, Khosla S, Jensen MD, Kirkland JL (2010). Fat tissue, aging, and cellular senescence. Aging Cell.

[3] Mattson MP (2010). Perspective: does brown fat protect against diseases of aging?. Ageing Res Rev.

[4] Woo J (2015). Obesity in older persons. Curr Opin Clin Nutr Metab Care.

[5] Decaria JE, Sharp C, Petrella RJ (2012). Scoping review report: obesity in older adults. Int J Obes.

[6] Hu FB, Meigs JB, Li TY, Rifai N, Manson JE (2004). Inflammatory markers and risk of developing type 2 diabetes in women. Diabetes.

[7] Pfannenberg C, Werner MK, Ripkens S, Stef I, Deckert A, Schmadl M, Reimold M, Häring HU, Claussen CD, Stefan N (2010). Impact of age on the relationships of brown adipose tissue with sex and adiposity in humans. Diabetes.

[8] Kajimura S, Spiegelman BM, Seale P (2015). Brown and beige fat: physiological roles beyond heat generation. Cell Metab.

[9] Zhang G, Li J, Purkayastha S, Tang Y, Zhang H, Yin Y, Li B, Liu G, Cai D (2013). Hypothalamic programming of systemic ageing involving IKK-β, NF-κ B and GnRH. Nature.

[10] Mobbs CV, Moreno CL, Poplawski M (2013). Metabolic mystery: aging, obesity, diabetes, and the ventromedial hypothalamus. Trends Endocrinol Metab.

[11] Shechter R, London A, Kuperman Y, Ronen A, Rolls A, Chen A, Schwartz M (2013). Hypothalamic neuronal toll-like receptor 2 protects against age-induced obesity. Sci Rep.

[12] Carrascosa JM, Ros M, Andrés A, Fernández-Agulló T, Arribas C (2009). Changes in the neuroendocrine control of energy homeostasis by adiposity signals during aging. Exp Gerontol.

[13] Spiegel S, Milstien S (2003). Sphingosine-1-phosphate: an enigmatic signalling lipid. Nat Rev Mol Cell Biol.

[14] Spiegel S, Milstien S (2011). The outs and the ins of sphingosine-1-phosphate in immunity. Nat Rev Immunol.

[15] Maceyka M, Harikumar KB, Milstien S, Spiegel S (2012). Sphingosine-1-phosphate signaling and its role in disease. Trends Cell Biol.

[16] Liu Y, Deng J, Wang L, Lee H, Armstrong B, Scuto A, Kowolik C, Weiss LM, Forman S, Yu H (2012). S1PR1 is an effective target to block STAT3 signaling in activated B cell-like diffuse large B-cell lymphoma. Blood.

[17] Liang J, Nagahashi M, Kim EY, Harikumar KB, Yamada A, Huang WC, Hait NC, Allegood JC, Price MM, Avni D, Takabe K, Kordula T, Milstien S, Spiegel S (2013). Sphingosine-1-phosphate links persistent STAT3 activation, chronic intestinal inflammation, and development of colitis-associated cancer. Cancer Cell.

[18] Theiss AL (2013). Sphingosine-1-phosphate: driver of NFκ B and STAT3 persistent activation in chronic intestinal inflammation and colitis-associated cancer. JAK-STAT.

[19] Nagahashi M, Hait NC, Maceyka M, Avni D, Takabe K, Milstien S, Spiegel S (2014). Sphingosine-1-phosphate in chronic intestinal inflammation and cancer. Adv Biol Regul.

[20] Lee H, Deng J, Kujawski M, Yang C, Liu Y, Herrmann A, Kortylewski M, Horne D, Somlo G, Forman S, Jove R, Yu H (2010). STAT3-induced S1PR1 expression is crucial for persistent STAT3 activation in tumors. Nat Med.

[21] Silva VR, Micheletti TO, Pimentel GD, Katashima CK, Lenhare L, Morari J, Mendes MC, Razolli DS, Rocha GZ, de Souza CT, Ryu D, Prada PO, Velloso LA (2014). Hypothalamic S1P/S1PR1 axis controls energy homeostasis. Nat Commun.

[22] Baranowski M, Charmas M, Długołęcka B, Górski J (2011). Exercise increases plasma levels of sphingoid base-1 phosphates in humans. Acta Physiol (Oxf).

[23] Baranowski M, Górski J, Klapcinska B, Waskiewicz Z, Sadowska-Krepa E (2014). Ultramarathon run markedly reduces plasma sphingosine-1-phosphate concen-tration. Int J Sport Nutr Exerc Metab.

[24] Baranowski M, Błachnio-Zabielska AU, Charmas M, Helge JW, Dela F, Książek M, Długołęcka B, Klusiewicz A, Chabowski A, Górski J (2015). Exercise increases sphingoid base-1-phosphate levels in human blood and skeletal muscle in a time- and intensity-dependent manner. Eur J Appl Physiol.

[25] Banitalebi E, Gharakhanlou R, Ghatrehsamani K, Parnow AH, Teimori H, Mohammad Amoli M (2013). The effect of resistance training on plasma S1P level and gene expression of S1P1,2,3 receptors in male Wistar rats. Minerva Endocrinol.

[26] Chiarreotto-Ropelle EC, Pauli LS, Katashima CK, Pimentel GD, Picardi PK, Silva VR, de_Souza CT, Prada PO, Cintra DE, Carvalheira JB, Ropelle ER, Pauli JR (2013). Acute exercise suppresses hypothalamic PTP1B protein level and improves insulin and leptin signaling in obese rats. Am J Physiol Endocrinol Metab.

[27] de_Moura LP, Souza Pauli LS, Cintra DE, de_Souza CT, da Silva AS, Marinho R, de_Melo MA, Ropelle ER, Pauli JR (2013). Acute exercise decreases PTP-1B protein level and improves insulin signaling in the liver of old rats. Immun Ageing.

[28] Hawley JA, Hargreaves M, Joyner MJ, Zierath JR (2014). Integrative biology of exercise. Cell.

[29] Ropelle ER, Flores MB, Cintra DE, Rocha GZ, Pauli JR, Morari J, de_Souza CT, Moraes JC, Prada PO, Guadagnini D, Marin RM, Oliveira AG, Augusto TM (2010). IL-6 and IL-10 anti-inflammatory activity links exercise to hypothalamic insulin and leptin sensitivity through IKKbeta and ER stress inhibition. PLoS Biol.

[30] Egan B, Zierath JR (2013). Exercise metabolism and the molecular regulation of skeletal muscle adaptation. Cell Metab.

[31] Allen J, Morelli V (2011). Aging and exercise. Clin Geriatr Med.

[32] Boström P, Wu J, Jedrychowski MP, Korde A, Ye L, Lo JC, Rasbach KA, Boström EA, Choi JH, Long JZ, Kajimura S, Zingaretti MC, Vind BF (2012). A PGC1-α-dependent myokine that drives brown-fat-like development of white fat and thermogenesis. Nature.

[33] Cadore EL, Pinto RS, Bottaro M, Izquierdo M (2014). Strength and endurance training prescription in healthy and frail elderly. Aging Dis.

[34] Strasser B (2013). Physical activity in obesity and metabolic syndrome. Ann N Y Acad Sci.

[35] Kaiyala KJ, Ramsay DS (2011). Direct animal calorimetry, the underused gold standard for quantifying the fire of life. Comp Biochem Physiol A Mol Integr Physiol.

[36] Samad F, Hester KD, Yang G, Hannun YA, Bielawski J (2006). Altered adipose and plasma sphingolipid metabolism in obesity: a potential mechanism for cardiovascular and metabolic risk. Diabetes.

[37] Kowalski GM, Carey AL, Selathurai A, Kingwell BA, Bruce CR (2013). Plasma sphingosine-1-phosphate is elevated in obesity. PLoS One.

[38] Błachnio-Zabielska AU, Pułka M, Baranowski M, Nikołajuk A, Zabielski P, Górska M, Górski J (2012). Ceramide metabolism is affected by obesity and diabetes in human adipose tissue. J Cell Physiol.

[39] Tran JR, Chen H, Zheng X, Zheng Y (2016). Lamin in inflammation and aging. Curr Opin Cell Biol.

[40] Wallet MA, Buford TW, Joseph AM, Sankuratri M, Leeuwenburgh C, Pahor M, Manini T, Sleasman JW, Goodenow MM (2015). Increased inflammation but similar physical composition and function in older-aged, HIV-1 infected subjects. BMC Immunol.

[41] Glass CK, Saijo K, Winner B, Marchetto MC, Gage FH (2010). Mechanisms underlying inflammation in neuro-degeneration. Cell.

[42] Shariat SF, Andrews B, Kattan MW, Kim J, Wheeler TM, Slawin KM (2001). Plasma levels of interleukin-6 and its soluble receptor are associated with prostate cancer progression and metastasis. Urology.

[43] Hobisch A, Eder IE, Putz T, Horninger W, Bartsch G, Klocker H, Culig Z (1998). Interleukin-6 regulates prostate-specific protein expression in prostate carcinoma cells by activation of the androgen receptor. Cancer Res.

[44] Chung TD, Yu JJ, Spiotto MT, Bartkowski M, Simons JW (1999). Characterization of the role of IL-6 in the progression of prostate cancer. Prostate.

[45] Nakashima J, Tachibana M, Horiguchi Y, Oya M, Ohigashi T, Asakura H, Murai M (2000). Serum interleukin 6 as a prognostic factor in patients with prostate cancer. Clin Cancer Res.

[46] Twillie DA, Eisenberger MA, Carducci MA, Hseih WS, Kim WY, Simons JW (1995). Interleukin-6: a candidate mediator of human prostate cancer morbidity. Urology.

[47] Gibala MJ, Gillen JB, Percival ME (2014). Physiological and health-related adaptations to low-volume interval training: influences of nutrition and sex. Sports Med.

[48] Gibala MJ, Little JP, Macdonald MJ, Hawley JA (2012). Physiological adaptations to low-volume, high-intensity interval training in health and disease. J Physiol.

[49] Hagobian TA, Yamashiro M, Hinkel-Lipsker J, Streder K, Evero N, Hackney T (2013). Effects of acute exercise on appetite hormones and ad libitum energy intake in men and women. Appl Physiol Nutr Metab.

[50] Schubert MM, Desbrow B, Sabapathy S, Leveritt M (2013). Acute exercise and subsequent energy intake. A meta-analysis Appetite.

[51] Thivel D, Isacco L, Montaurier C, Boirie Y, Duché P, Morio B (2012). The 24-h energy intake of obese adolescents is spontaneously reduced after intensive exercise: a randomized controlled trial in calorimetric chambers. PLoS One.

[52] Holmstrup ME, Fairchild TJ, Keslacy S, Weinstock RS, Kanaley JA (2013). Satiety, but not total PYY, Is increased with continuous and intermittent exercise. Obesity (Silver Spring).

[53] Borer KT, Wuorinen E, Ku K, Burant C (2009). Appetite responds to changes in meal content, whereas ghrelin, leptin, and insulin track changes in energy availability. J Clin Endocrinol Metab.

[54] Pedersen BK, Febbraio MA (2012). Muscles, exercise and obesity: skeletal muscle as a secretory organ. Nat Rev Endocrinol.

[55] Besse-Patin A, Montastier E, Vinel C, Castan-Laurell I, Louche K, Dray C, Daviaud D, Mir L, Marques MA, Thalamas C, Valet P, Langin D, Moro C, Viguerie N (2014). Effect of endurance training on skeletal muscle myokine expression in obese men: identification of apelin as a novel myokine. Int J Obes.

[56] Aoi W, Naito Y, Takagi T, Tanimura Y, Takanami Y, Kawai Y, Sakuma K, Hang LP, Mizushima K, Hirai Y, Koyama R, Wada S, Higashi A (2013). A novel myokine, secreted protein acidic and rich in cysteine (SPARC), suppresses colon tumorigenesis via regular exercise. Gut.

[57] Botelho M, Cavadas C (2015). Neuropeptide Y: an anti-aging player?. Trends Neurosci.

[58] Fernández-Galaz C, Fernández-Agulló T, Campoy F, Arribas C, Gallardo N, Andrés A, Ros M, Carrascosa JM (2001). Decreased leptin uptake in hypothalamic nuclei with ageing in Wistar rats. J Endocrinol.

[59] El-Haschimi K, Pierroz DD, Hileman SM, Bjørbaek C, Flier JS (2000). Two defects contribute to hypothalamic leptin resistance in mice with diet-induced obesity. J Clin Invest.

[60] Bence KK, Delibegovic M, Xue B, Gorgun CZ, Hotamisligil GS, Neel BG, Kahn BB (2006). Neuronal PTP1B regulates body weight, adiposity and leptin action. Nat Med.

[61] Münzberg H, Huo L, Nillni EA, Hollenberg AN, Bjørbaek C (2003). Role of signal transducer and activator of transcription 3 in regulation of hypothalamic proopiomelanocortin gene expression by leptin. Endocrinology.

[62] Lebedev SV, Blinov DV, Petrov SV (2004). Spatial characteristics of cisterna magna in rats and novel technique for puncture with a stereotactic manipulator. Bull Exp Biol Med.

[63] Chibalin AV, Yu M, Ryder JW, Song XM, Galuska D, Krook A, Wallberg-Henriksson H, Zierath JR (2000). Exercise-induced changes in expression and activity of proteins involved in insulin signal transduction in skeletal muscle: differential effects on insulin-receptor substrates 1 and 2. Proc Natl Acad Sci USA.

