# Correction for: Hypothalamic S1P/S1PR1 axis controls energy homeostasis in Middle-Aged Rodents: the reversal effects of physical exercise

**DOI:** 10.18632/aging.103194

**Published:** 2020-05-30

**Authors:** Vagner Ramon Rodrigues Silva, Carlos Kiyoshi Katashima, Carla G. Bueno Silva, Luciene Lenhare, Thayana Oliveira Micheletti, Rafael Ludemann Camargo, Ana Carolina Ghezzi, Juliana Alves Camargo, Alexandre Moura Assis, Natalia Tobar, Joseane Morari, Daniela S. Razolli, Leandro Pereira Moura, José Rodrigo Pauli, Dennys Esper Cintra, Lício Augusto Velloso, Mario J.A. Saad, Eduardo Rochete Ropelle

**Affiliations:** 1 School of Applied Sciences, University of Campinas, Limeira, SP, Brazil; 2 Department of Internal Medicine, University of Campinas, Campinas, SP, Brazil; 3 CEPECE - Research Center of Sport Sciences, School of Applied Sciences, University of Campinas, Limeira, SP, Brazil; 4 Laboratory of Cell Signaling, Obesity and Comorbidities Research Center, University of Campinas, Campinas, 1308-970, Brazil

**Keywords:** correction

Original article: Aging. 2017; 9:142–155.  . https://doi.org/10.18632/aging.101138

**This article has been corrected:** The authors requested replacement of Figure 4h (upper panel). Originally, to represent the morphology of brown adipose tissue (BAT) in the Middle-Aged group, the authors used the same image in Figure 3c (lower panel) and Figure 4h (upper panel). To avoid misinterpretation, they have replaced the Middle-Aged, Rest panel (Figure 4h) with a representative image from the original set of experiments. This alteration does not affect the results or conclusions drawn in this work.

**Figure 4:**
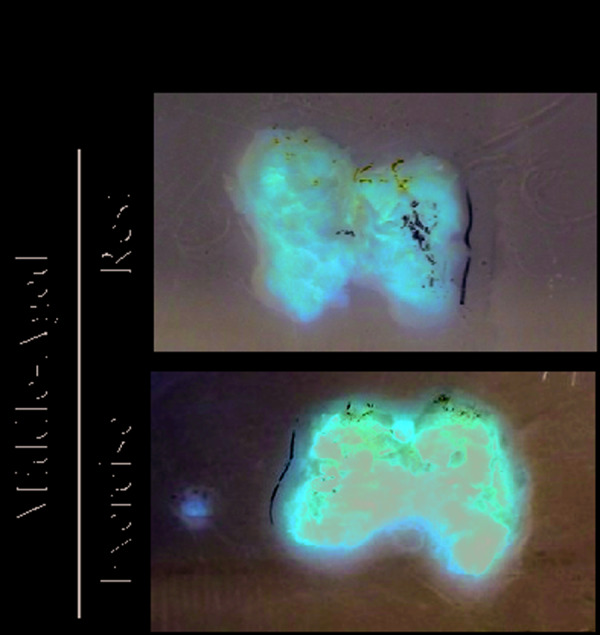
**Acute exercise improves S1PR1/STAT3 in the hypothalamus of rats.** Western blot showing UCP1 expression in BAT (**g**) and Image of BAT (n=6 per group) (**h**).

