# Supplementary material for: Hypothalamic S1P/S1PR1 axis controls energy homeostasis in Middle-Aged Rodents: the reversal effects of physical exercise
Source: Aging (Albany NY). 2016 Dec 26;9(1):142–54. doi: 10.18632/aging.101138 (PMC5310661; doi:10.18632/aging.101138)
Supplement: Supplementary file 1 [file aging-09-0142-s001.pdf]

SUPPLEMENTARY MATERIALS

Please browse the links in the **Full Text** version of this manuscript to see Supplementary 4 with the membranes uncut.

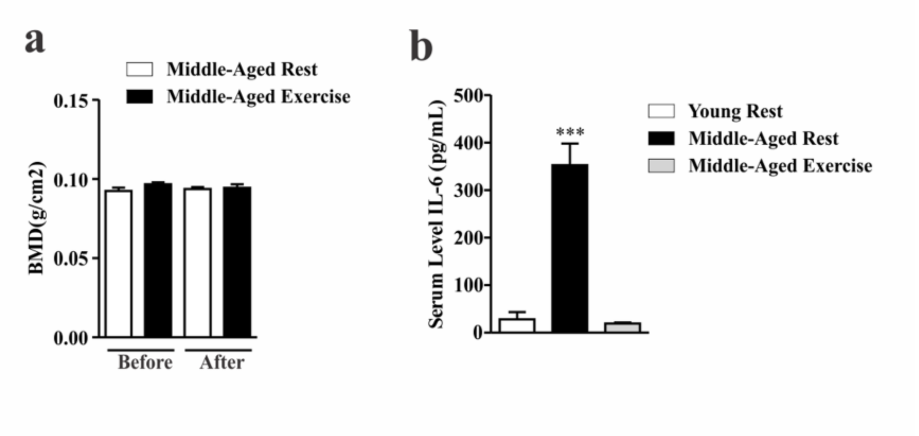

Supplementary Figure S1. Serum IL-6 level and Bone Mineral Density after chronic exercise in middle-aged mice. Dual energy X-ray absorptiometry analyses was performed to evaluate the bone Mineral Density (BMD) **(a)** (n=3 per group). Samples for analyses of serum IL-6 levels were collected after placing mice through the chronic exercise protocol for mice **(b)** (n=5–6 per group). One-way ANOVA was used for analyses where (b) *** p<0.0001 vs Young Rest and Middle-Aged Exercised.

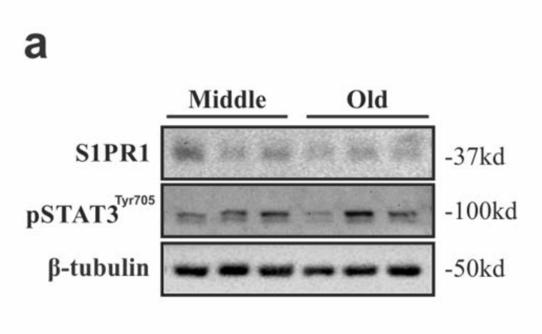

Supplementary Figure S2. Western blot analysis in the hypothalamus of old rats. Western blot showing in the protein level of S1PR1 and STAT3 phosphorylation hypothalamus of Middle-Aged and old Wistar rats. (n=6 per group).

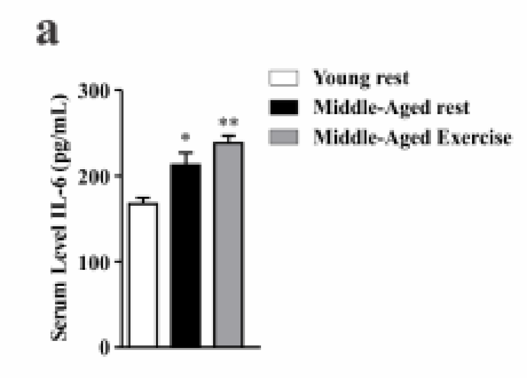

Supplementary Figure S3. Serum level of IL-6 in middle-aged rats after acute exercise. Samples were collected following the acute protocol exercise for rats (n=4 per group). One-way ANOVA was performed to (a) **p<0.05 vs young rest and * p<0.05 vs young rest.
